# Trimethylamine N-oxide and kidney diseases: what do we know?

**DOI:** 10.1590/2175-8239-JBN-2023-0065en

**Published:** 2023-12-01

**Authors:** Ozkan Gungor, Nuri Baris Hasbal, Demet Alaygut

**Affiliations:** 1 Kahramanmaras Sutcu Imam University, School of Medicine, Department of Internal Medicine, Kahramanmaras, Turkey.; 2 Koc University, School of Medicine, Department of Internal Medicine, Istanbul, Turkey.; 3 Izmir Katip Celebi University, School of Medicine, Department of Pediatrics, Izmir, Turkey.

**Keywords:** Trimethylamine N-oxide, Gut Microbiota, Kidney Disease, N-óxido de Trimetilamina, Microbiota Intestinal, Doença Renal

## Abstract

In the human gut, there is a metabolically active microbiome whose metabolic products reach various organs and are used in the physiological activities of the body. When dysbiosis of intestinal microbial homeostasis occurs, pathogenic metabolites may increase and one of them is trimethyl amine-N-oxide (TMAO). TMAO is thought to have a role in the pathogenesis of insulin resistance, diabetes, hyperlipidemia, atherosclerotic heart diseases, and cerebrovascular events. TMAO level is also associated with renal inflammation, fibrosis, acute kidney injury, diabetic kidney disease, and chronic kidney disease. In this review, the effect of TMAO on various kidney diseases is discussed.

## Resumo

No intestino humano, existe um microbioma metabolicamente ativo cujos produtos metabólicos alcançam diversos órgãos e são utilizados nas atividades fisiológicas do corpo. Quando ocorre disbiose da homeostase microbiana intestinal, os metabólitos patogênicos podem aumentar, e um deles é o N-óxido de trimetilamina (TMAO). Acredita-se que o TMAO tenha um papel na patogênese da resistência à insulina, diabetes, hiperlipidemia, doenças cardíacas ateroscleróticas e eventos cerebrovasculares. O nível de TMAO também está associado à inflamação renal, fibrose, lesão renal aguda, doença renal diabética e doença renal crônica. Nesta revisão, discute-se o efeito do TMAO em diversas doenças renais.

## Introduction

The human gut contains a complex and metabolically active microbial ecosystem named microbiota. Although the microbiota is vital, changes in its composition and function may induce metabolic processes that may lead to changes in host phenotypes. Metabolites of the gut microbiota reach various tissues and organs through blood circulation and are used in the physiological activities of the host. In this context, the gut microbiota is an important organ for the host, as it plays a key role in maintaining the integrity of the mucosal barrier and immune system regulation^1^. However, it connects the gut, liver, brain, and other organs through host-microbiota joint metabolism to form metabolic axes that regulate the host’s systemic metabolism. In case of dysbiosis of intestinal microbial homeostasis, metabolites may increase and cause several diseases. Trimethyl amine-N-oxide (TMAO), one of these metabolites, has been the subject of numerous studies in recent years.

Trimethylamine (TMA) is produced by the intestinal microflora with the metabolism of phosphatidylcholine/choline, carnitine, betaine, dimethylglycine, and ergothioneine^2^. TMA is absorbed into the bloodstream and converted to TMAO by hepatic flavin monooxygenases (FMO1 and FMO3) and microbial metabolism^3^. The highest amounts of TMAO in food are found in saltwater fish, which contain about 3 g/kg of this compound^4^. Food products rich in phosphatidylcholine considered the main dietary source of choline and therefore TMAO are eggs, liver, milk, meat, and fish^5^. After absorption, most of the TMA (about 95%) is oxidized to TMAO, which is transported to tissues for accumulation as osmolyte or, more often, cleared by the kidneys^2,4^.

**Figure 1.**
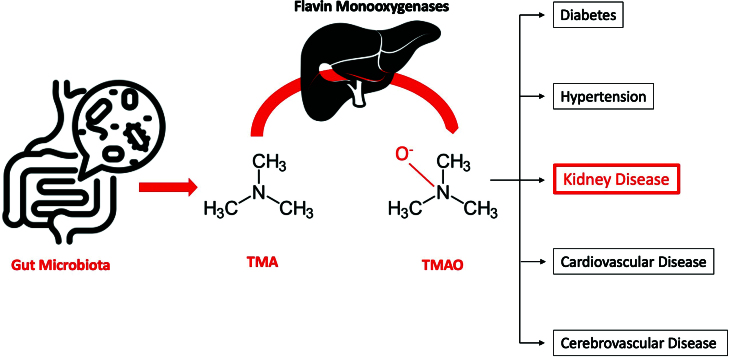
TMAO and its associations with various diseases.

Although TMAO has been known for a long time, Wang et al.^6^ suggested that TMAO may be harmful to human health. An increase in TMAO concentration may result from diet, changes in the composition of the intestinal microbiota, intestinal dysbiosis, or disruption of the intestinal barrier. TMAO is thought to contribute to the pathogenesis of hypertension, diabetes, atherosclerotic heart disease, and neurological diseases (Figure 1). TMAO level is associated with impaired kidney function^7^. Serum concentrations of TMAO and TMA are increased in patients with reduced kidney function, such as hemodialysis (HD) patients, compared to healthy subjects.

Patients with kidney diseases have a decreased capacity for systemic metabolite clearance and are at high risk of accumulating all kinds of intestinal microbial metabolites^8^. Uremic toxins derived from the intestinal microbiota stimulate adverse pathophysiological features that play a role in the progression of chronic kidney disease and create independent risk factors for changes in the kidneys, including fibrosis, nephron loss, reduction in tubular function and glomerular filtration, and cardiovascular disease (CVD)^9^. In the Framingham Heart Study, the metabolomic profiling of 1434 people with a basal eGFR value ≥60 mL/1.73 m^2^, identified 9 metabolites that affect the development of CKD, and circulating choline, the precursor of TMAO, was one of them^10^. Mammals cannot metabolize TMAO, and 95% of it is excreted unchanged from the body via glomerular filtration and tubular secretion^11,12^. It has been reported that TMAO concentration is negatively correlated with GFR (measured) and is approximately thirty times higher in HD patients compared to healthy individuals^8,13^. Tang et al.^14^ also showed that high TMAO concentrations were associated with 2.8-fold increased mortality, in addition to a reduction in renal function.

Some studies reported that TMAO is associated with acute kidney injury, renal inflammation, and fibrosis. In this review, the effect of TMAO on CKD, hypertension, diabetic kidney disease (DKD), and kidney transplantation will be discussed.

### TMAO and Chronic Kidney Disease

TMAO has been associated with kidney diseases by two important observations: patients with CKD have high plasma TMAO concentration and TMAO induce kidney disease in experimental animal models. Circulating TMAO concentrations start to increase when eGFR declines below 60 mL/min/ 1.73 m^2 15^. Increased TMAO concentrations are associated with systemic inflammatory markers and reduced 5-year survival^16^. Decreasing TMAO levels is possible with hemodialysis (HD), and kidney transplantation also appears to improve TMAO levels^17^.

C57BL/6J mice fed a high-choline diet or TMAO for six weeks showed increased plasma concentrations of TMAO associated with increases in tubulointerstitial fibrosis, collagen deposition, and kidney injury marker-1^14^. Mice were fed this way for 16 weeks also had increased serum cystatin C concentrations. Although TMAO levels are high in those with renal disease, there is no definite and sufficient evidence showing that TMAO causes kidney disease. Since plasma concentration of TMAO is determined by renal clearance and kidney function, TMAO levels does not increase without a decline in kidney function. The Comprehensive Dialysis Study, which prospectively evaluated 235 patients with ESRD who underwent HD and peritoneal dialysis (PD), found no association between serum TMAO levels and mortality and cardiovascular outcomes^18^. In a study investigating the relationship between TMAO levels and hospitalization, 69 HD outpatients were evaluated^19^. The patients were divided into two groups according to high and low TMAO levels. The risk of hospitalization was found to be higher in patients with high TMAO levels. In both groups, arteriovenous fistula dysfunction and cardiovascular diseases were two significant causes of hospitalization. At one-year long-term follow-up, vascular dysfunction was more common in those with high TMAO levels, but there was no difference in hypertension, diabetes mellitus, interdialytic hypotension, calcium, phosphorus, PTH, and LDL.

Like HD, PD patients have higher TMAO levels than the healthy population. In PD patients, high TMAO levels are associated with peritonitis^20^. With the disruption of the original intestinal flora and an increase of a more pathogenic microbiota, intestinal dysbiosis and increased urea weaken the intestinal barrier function, increasing the host’s susceptibility to pathogen invasion^21^.

Although TMAO level is elevated secondary to decreased eGFR in patients with ESRD, TMAO may also be released from the renal medulla due to ischemic kidney damage^22^. Elevated plasma TMAO levels are associated with poor prognosis in CKD. In a mouse model of CKD, TMA formation was suppressed after feeding with an indirect TMAO inhibitor, iodomethylcholine, and kidney injury molecules and cystatin C levels were decreased^23^. Histopathological examination of mice with increased TMAO levels revealed tubulointerstitial fibrosis and collagen deposition. This finding indicated that TMAO levels play a role in the development and progression of CKD.

Histopathologically, the presence of myofibroblasts is a prognostic index for development and progression of fibrosis and progression of tubular atrophy^22^. Studies have reported several molecular biomarkers that may be associated with tubulointerstitial fibrosis. The NLRP3 inflammasome has been shown to play a role in the development of fibrosis in many diseases^24^. In kidney disease, it has been particularly examined in the progression of acute kidney injury, chronic kidney disease, and diabetic nephropathy. A study examining the effect of TMAO on renal fibrosis found that TMAO promotes the activation of renal fibroblasts by increasing α-smooth muscle actin levels. Resident fibroblasts of the renal interstitium differentiate into myofibroblasts in response to some growth factors (such as TGF-ß1, FGF, IL-1, PDGF, TNF-α, and aldosterone)^25^. TMAO is a potent renal fibroblast activator. It can promote fibroblast proliferation equivalent to TGF-ß1 activation. It increases the production of total collagen from renal fibroblasts but does not affect fibronectin or TGF-ß1. In other words, TMAO does not exert its fibrotic effect by releasing TGF-ß1^22^. Kapetanaki et al.^22^ showed that TMAO exerts its fibrotic effect by using renal fibroblasts’ PERK/Akt /mTOR pathway, NLRP3, and caspase 1 signals. TMAO activates protein kinase R-like endoplasmic reticulum kinase (PERK), an endoplasmic reticulum (ER) stress kinase found in hepatocytes, by directly binding to it. Identification of PERK as a receptor of TMAO suggests that PERK inhibition can reduce TMAO-associated collagen production and proliferation of renal fibroblasts.

Plasma TMAO levels are positively correlated with atherosclerotic plaques in the aorta. Imbalance in the intestinal microbiota leads to CVD through inflammation and oxidative stress. Patients with CKD presented with accumulation of uremic toxins such as p-cresyl sulfate and indoxyl sulfate resulting from amino acid degradation, and these uremic toxins were found to be associated with CVD mortality^26,27^. In recent years, TMAO has been regarded as a proatherogenic metabolite^15^. Animal studies have shown that TMAO administration is correlated with plaque size^6^. High L-carnitine levels in patients undergoing cardiac evaluation were found to be correlated with high TMAO levels and associated with CVD, myocardial infarction, stroke, and death^15^.

A study examining 115 children and adolescents with CKD stages 1–4 found plasma TMAO, TMA, and dimethylamine levels to be high in those with CKD stages 2–4. In 53% of these children, BP load and ambulatory arterial stiffness index, another CVD risk indicator, increased significantly during 24-hour ambulatory BP follow-up^28^. TMAO worsens existing heart failure and increases the hypertensive effect of angiotensin II^29^. Endothelial dysfunction is a key factor in the pathogenesis of cardiovascular diseases. Evaluation of endothelial dysfunction in a rat model of CKD revealed that circulating TMAO levels were increased in rats with CKD, and this could be prevented in rats treated with 1% 3,3-dimethyl-1-butanol (DMB). Endothelium-dependent vasodilation was impaired in these rats and improved with DMB treatment. Vascular eNOS activity was decreased, superoxide production and pro-inflammatory cytokines were increased, and DMB treatment normalized vascular eNOS activity, superoxide production, and proinflammatory cytokines. All these findings show that increased circulating TMAO could lead to vascular oxidative stress and inflammation, resulting in endothelial dysfunction^29^. New treatment strategies that can be developed by targeting TMAO are of great importance for the prevention and treatment of CVD secondary to CKD.

### TMAO and Hypertension

**Figure 2.**
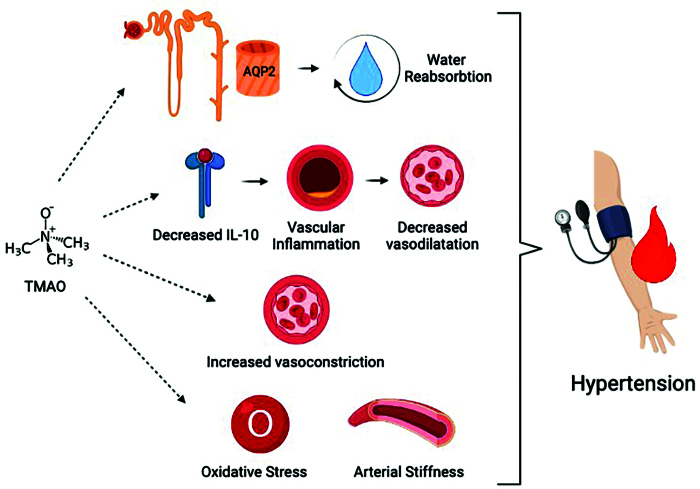
Relationship between TMAO and hypertension.

The number of human and animal studies on the relationship between TMAO and hypertension is limited. Although no study has shown this relationship directly, considering the data obtained from some animal studies and the effects of TMAO on CV morbidity and mortality, its indirect effect may be possible. In a study by Liu et al^30^ on hypertensive mice, the authors suggested that increased circulating TMAO levels increased the expression of aquaporin-2 in the renal medulla and increased plasma osmotic pressure, leading to more water reabsorption, thus causing an increase in blood pressure. There are also studies suggesting that increased circulating TMAO level is associated with increased vascular inflammation, decreased vasodilation, and hypertension by decreasing interleukin-10 levels^31^. It has also been shown that TMAO increases angiotensin-2-related vasoconstriction, thereby increasing angiotensin-2-related hypertension^32^. Controversial results have been presented in human studies reflecting the relationship between TMAO and hypertension. In a study evaluating microbial metabolites in feces and serum and TMAO levels in serum, it was demonstrated that serum TMAO levels were not different in hypertensive and normotensive individuals, but other trials found that high serum TMAO levels are associated with higher prevalence of hypertension^33,34^. Another important evidence is that the level of circulating TMAO is higher in hypertensive people than in normotensive ones, and higher in hypertensive people with renal dysfunction than in hypertensive ones without renal dysfunction^35^. It should not be underestimated that the level of circulating TMAO is affected by many different factors, especially diet and co-morbidities. Brunt et al.^36^ showed that the level of circulating TMAO increases with aging and, accordingly, TMAO affects the formation of aortic stiffness and hypertension through the accumulation of advanced glycation end products and increase in oxidative stress. Considering all this evidence, the relationship between TMAO and hypertension has become increasingly clear, and TMAO has become a target molecule in the treatment of hypertension. The relationship between hypertension and TMAO is shown in Figure 2.

### TMAO and Diabetic Kidney Disease

The relationship between DKD and TMAO is not yet clear. However, the negative effects of TMAO on endothelial dysfunction, atherosclerosis, inflammation, and oxidative stress have led to a hypothesis that it is also associated in DKD with a similar pathophysiological mechanism. An increase in renal dysfunction and fibrosis was detected in mice given TMAO, while structural and functional improvement was observed when agents that inhibit TMAO formation were given in an animal model^37^. In rats with a DKD model induced by streptozotocin, Fang et al.^38^ demonstrated that plasma TMAO levels were high in rats with DKD, and they showed worsening of kidney functions, fibrosis, and inflammation, and an increase in IL-1b and IL-18 secretion were observed with the administration of TMAO. In a study including 1159 patients with type 1 diabetes, high plasma TMAO level was predictive of mortality and cardiovascular and renal endpoints but was not associated with progression of DKD^39^. In another trial in which 555 patients with DKD were evaluated, plasma TMAO level was a determinant for death from all causes, and low urine/plasma TMAO ratio was also associated with death due to CV causes^40^. In addition, Yang et al.^41^, in a controlled study conducted with a small cohort, showed that serum TMAO level is high in individuals with DKD and that there is a positive correlation between serum TMAO level and albuminuria, and they claimed that TMAO may be a marker for DKD. In light of all these data, it is suggested that the circulating TMAO level can be both an indicator and a treatment target in DKD. However, larger, randomized, controlled, and well-designed studies are needed.

### TMAO and Kidney Transplantation

Kidney transplantation causes a complex metabolic situation due to the presence of a foreign organ and the immunosuppressive agents used to maintain the function of this organ. Naturally, the relationship between TMAO and transplantation raises interest. In a prospective, controlled study by Poesen et al.^42^, in which 51 patients with kidney transplants were included, a significant decrease was observed in serum TMAO levels just after kidney transplantation, but it was determined that there was no significant difference between TMAO levels in kidney transplant patients and CKD patients as the control group at the third month and first year post-transplant. In another study, despite the limited number of patients and no control group, there was a significant decrease in plasma TMAO levels in the third month after the transplantation (71.3 mM vs. 11.1 mM)^17^. Therefore, it may be assumed that kidney transplantation reduces serum TMAO levels in the early period, but its effect in the follow-up period is not clear. In addition, there is no study evaluating whether there is a relationship between serum TMAO level and survival in this patient group. However, Flores-Guerrero et al.^43^ showed an independent positive correlation between serum TMAO level and risk of graft failure. In another study investigating the use of calcineurin inhibitors, a relationship was found between serum TMAO levels and high cyclosporine levels, but no similar relationship was observed with tacrolimus, so a possible relationship between TMAO level and calcineurin inhibitor toxicity can be mentioned^44^. Again, there is a need for more extensive research on the relationship between kidney transplantation and TMAO.

### Tmao and Other Conditions

The search for new markers has been one of the most studied areas of research, because of the inadequacy of classical indicators for early detection of acute kidney injury (AKI). However, no study has yet been published that clearly shows the relationship between AKI and TMAO. In a study in which André et al.^45^ applied the spectrometric method, which allows the evaluation of seven uremic toxins in AKI, they showed that TMAO accumulation was not excessive. In another study evaluating the effect of physical activity on AKI indicators, a statistically significant increase was observed in urea and creatinine values after significant physical activity (10 and 100 km running), while no significant increase was observed in serum TMAO levels^46^. Limited and insufficient evidence shows no significant relationship between AKI and TMAO, but further studies are needed.

In a study evaluating nine cardiovascular drug groups, loop diuretics were shown to increase plasma levels of TMAO by decreasing urinary excretion; therefore, it may be recommended that the use of loop diuretics should not be ignored in human studies^47^.

In conclusion, recent evidence suggests that TMAO plays a role in the pathogenesis of renal inflammation, fibrosis, hypertension, diabetic kidney disease, acute kidney injury, and chronic kidney disease. Further well-designed human studies are extremely needed.
